# Efficacy and Safety of JAK1 Inhibitor Abrocitinib in Atopic Dermatitis

**DOI:** 10.3390/pharmaceutics15020385

**Published:** 2023-01-23

**Authors:** Helena Iznardo, Esther Roé, Esther Serra-Baldrich, Lluís Puig

**Affiliations:** 1 Dermatology Department, Hospital de la Santa Creu i Sant Pau, Mas Casanovas 90, 08041 Barcelona, Spain; 2 Institut d’Investigació Biomèdica Sant Pau (IIB SANT PAU), 08041 Barcelona, Spain; 3 Sant Pau Teaching Unit, School of Medicine, Universitat Autònoma de Barcelona, 08041 Barcelona, Spain

**Keywords:** abrocitinib, jak inhibitor, JAK1, atopic dermatitis

## Abstract

Abrocitinib is a JAK1 selective inhibitor recently approved for the treatment of moderate-to-severe atopic dermatitis in adults. It has demonstrated efficacy and safety in several clinical trials, both in children and adults, in monotherapy, and compared with dupilumab. The expected EASI-75 response rate estimates at week 12 are 62.9% (95% CrI 42.5–79.9%) for abrocitinib 200 mg and 43.0% (95% CrI 24.8–64.0%) for abrocitinib 100 mg. Abrocitinib has shown a faster effect than dupilumab as regards early alleviation of itch. Because of the incomplete target selectivity of JAK inhibitors, when abrocitinib treatment is considered, laboratory screening is necessary, latent tuberculosis must be screened for, active infections are a contraindication, and special caution must be exerted in treating elderly patients and those predisposed to thromboembolic events. Even though recent meta-analyses of clinical trials have not shown that atopic dermatitis, or its treatment with JAK inhibitors or dupilumab, modify the risk of deep venous thrombosis or pulmonary embolism, long-term follow-up studies will better define the safety profile of abrocitinib.

## 1. Introduction

Atopic dermatitis (AD) is a chronic immune-mediated inflammatory skin condition with a high prevalence in both children and adults [1]. It is characterized by intensely itching and inflammatory eczematous lesions, and its course can be continuous or relapsing-resolving with repeated flare-ups [1]. The prevalence of AD has increased significantly in the past 30 years, with an estimated 10–20% in developed countries [2]. AD starts in early childhood in almost 60% of cases, although it can manifest at any age [3]. A protracted and severe disease course is more likely in patients with early and severe onset, a family history of AD, and early sensitization to allergens [1]. AD carries a significant health burden, and symptoms such as itch and sleep deprivation ostensibly affect the quality of life of both patients and relatives. Moreover, AD comorbidities (asthma, allergic sensitization, depression, anxiety, and other neurodevelopmental issues such as attention-deficit disorder) also have an important economic effect on society [1]. 

**Figure 1:**
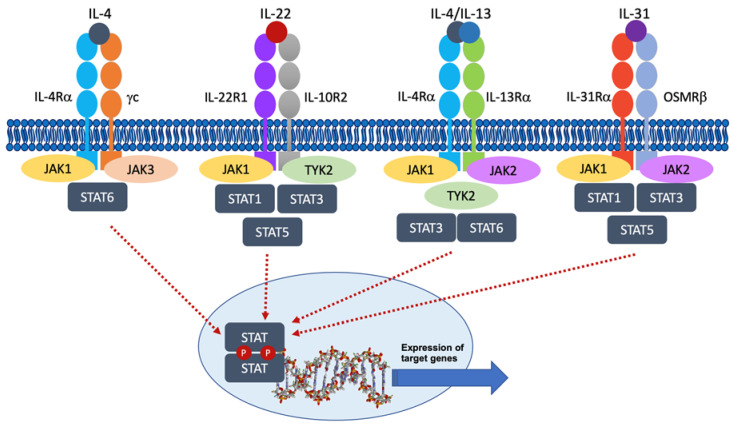
JAK/STAT signaling pathway in atopic dermatitis. Activation of the respective JAKs upon cytokine binding to their cognate receptor induces phosphorylation of STATs and their dimerization, translocating to the nucleus and regulating gene transcription. IL: interleukin; JAK: Janus kinase; STAT: signal transducer and activation of transcription.

The pathogenesis of AD is complex and involves two mutually reinforcing mechanisms: epidermal barrier disruptions in structure and function and cutaneous inflammation due to inappropriate immune responses [1]. Acute cutaneous inflammation in AD is characterized by a Th2-dominated response involving IL-4, IL-13, and IL-33. In chronic lesions, interferon-gamma (IFN-**γ**), IL-17, and IL-22 are also important [4]. Many cytokines involved in skin inflammation signal through type I (with a conserved extracellular domain that contains a WSXWS consensus motif) and II (for interferons and IL-10 family members) cytokine receptors. These receptors lack intrinsic kinase activity and depend on associated protein kinases to signal within the cell upon stimulation, the Janus kinase (JAK) family [4], constituted by JAK1, JAK2, JAK3, and tyrosine kinase 2 (TYK2) [5]. JAK 1, JAK 2, and TYK2 are expressed in multiple cell types, whereas JAK3 is expressed primarily in hematopoietic cells [6]. JAKs association with the intracellular domain of cytokine receptors is facilitated by N-terminal FERM and SH2-related domains, whereas kinase domains allow JAKs to phosphorylate proteins, and pseudokinase domains are regulatory in nature. The binding of ligands to their cell surface receptors causes them to dimerize, thus bringing the associated JAKs close together. JAKs phosphorylate each other on tyrosine residues, increasing the activity of their kinase domains, and then phosphorylate tyrosine residues on the receptor subunits and create binding sites for the SH2 domains of intracytoplasmic transcription factors known as signal transducer and activator of transcription (STAT) [7,8]. STATs are then phosphorylated by JAKs, become dissociated, and form hetero- or homodimers, characterized by binding of the SH2 domain of each STAT to the phosphorylated tyrosine of the other, and translocate into the nucleus to regulate target gene expression of inflammatory mediators [7]. To date, more than 50 cytokines signaling via the JAK/STAT pathway have been identified, and each JAK receptor may be associated with multiple cytokine receptors, having different downstream effects [9]. JAK-STAT is also a common signal transduction pathway involved in cell proliferation, migration, differentiation, and apoptosis [4,10]. All these factors explain why JAK inhibitors have broader immune-suppressing effects than monoclonal antibodies [11] (Figure 1). 

Abrocitinib (PF-04965842) (Cibinqo^®^, Pfizer, New York, NY, USA) is a small molecule that can be orally administered and selectively inhibits JAK1 protein. It is currently approved for the treatment of moderate-to-severe AD in adults by the European Medicines Agency (EMA) and the United States Food and Drug Administration (FDA). In this narrative review, we will discuss the relevant literature about abrocitinib for AD, with consideration to its efficacy and safety data, especially regarding monitoring and immunosuppression concerns.

## 2. Methods

A PubMed search with the term “abrocitinib” yielded 117 results; all the abstracts were screened, and the papers considered more important by the authors, as well as relevant general references on JAKs, were included in this review.

## 3. JAK1 Inhibition

JAK inhibitors are small molecules and can be classified into different categories according to their relative selectivity: non-selective or pan-JAK inhibitors (delgocitinib, cerdulatinib, jaktinib); dual inhibitors (baricitinib [JAK1/JAK2], ruxolitinib [JAK1/JAK2], brepocitinib [JAK1/TYK2], ATI-1777 [JAK1/JAK3]) and selective JAK1 inhibitors (upadacitinib, abrocitinib, ivarmacitinib) [12]. In preclinical studies, abrocitinib showed 28-fold selectivity for JAK1 over JAK2, >340-fold selectivity over JAK3, and 43-fold selectivity over TYK2 [13]. Signaling cytokines mediated by JAK1 include IL-4, IL-13, and IL-31, all involved in AD pathogenesis (Figure 1) [4]. JAK1 is also associated with many AD-relevant cytokines: type II cytokine receptors for IL-10, IL-19, IL-20, and IL-22, as well as glycoprotein 130 (gp130) – a shared subunit of several type I cytokine receptors, including IL-6 and IL-12 –, signal primarily through JAK1, but also associate with JAK2 and TYK2 [14]; IFN-α and INF-β signal through a combination of JAK1 and TYK2, and the IFN-γ receptor activates both JAK1 and JAK2 [15]. Finally, receptors for IL-2, IL-4, IL-7, IL-9, IL-13, IL-15, and IL-21 signal with JAK1 and JAK3 [15].

## 4. Abrocitinib: Pharmacokinetics and Pharmacodynamics

Oral absorption of abrocitinib is 91%, and food intake does not influence it [16]. Peak plasma concentrations are reached within 1 h of oral intake, the half-life is 5 h, and steady-state plasma concentrations are reached within 48 h when abrocitinib is taken once daily [16]. There are three major metabolites (pyrrolidinone pyrimidine and 2-hydroxypropyl) and a minor metabolite (3-hydroxypropyl); the first is inactive, and the latter two are active. Abrocitinib is metabolized mainly by CYP2C19 (~53%) and CYP2C9 (~30%) and to a lesser extent by CYP3A4 (~11%) and CYP2B6 (~6%) [16]. One clinical trial assessing the effects of CYP2C19 inhibition or induction on abrocitinib pharmacokinetics and pharmacodynamics found that when administered with strong CYP2C19 inhibitors, the dose should be reduced by half, but an adjustment is not required when administered with strong CYP2C19/2C9 inducers [17].

The effects of alterations in hepatic and renal functions have been evaluated in phase 1 clinical trials [18,19]. In one open-label, nonrandomized, single-dose study, 200 mg of abrocitinib was administered to patients with varying degrees of liver function (normal, mild impairment, and moderate impairment) [18]. No clinically relevant effects on pharmacokinetics were found, and dose adjustments were not needed according to liver function. Patients with severe hepatic impairment were not included in this study [18]. The same design was used for renal function: patients with moderate and severe renal impairment had increased levels of abrocitinib and active metabolites [19]. A dose reduction by half in patients with severely decreased renal function is advised [19]. Furthermore, abrocitinib is contraindicated in patients with severe hepatic impairment and patients with end-stage renal disease on dialysis.

Reduction of platelets peaks at 4 weeks after starting abrocitinib and then returns to normal levels throughout the rest of the treatment. The pathogenic mechanism for this reduction is not yet understood but could be related to decreased JAK1 activity resulting in decreased downstream thrombopoietin production [20]. Dose-dependent increases in low-density lipoprotein (LDL) cholesterol, high-density lipoprotein (HDL) cholesterol, and total cholesterol levels have been observed following 4 weeks of abrocitinib treatment. Abrocitinib 100 mg resulted in a 10% increase in LDL cholesterol, while 200 mg resulted in a 15% increase [21]. 

## 5. Abrocitinib: Efficacy and Safety Data

Abrocitinib has shown efficacy in patients with moderate-to-severe AD in monotherapy (MONO-1 and 2 clinical trials) as well as in combination with topical therapies in comparison with placebo (COMPARE clinical trial); furthermore, efficacy has also been demonstrated in adolescents in combination with topical therapies (JADE TEEN clinical trial) [22,23,24,25,26,27,28,29]. The available results of the clinical trials are summarized in Table 1.

In the phase 1 study, safety, tolerability, pharmacokinetics, and pharmacodynamics of multiple ascending doses of abrocitinib were evaluated in 79 healthy individuals. The most frequent treatment-related adverse events (AEs) were headaches (16.4%), diarrhea (13.9%), and nausea (13.9%). No deaths or serious AEs were reported [30].

The efficacy and safety of multiple doses of abrocitinib in 267 adult patients with moderate-to-severe AD were assessed in a phase 2b clinical trial [22]. By week 12, clear (0) or almost clear (1) Investigator’s Global Assessment (IGA) scores, with an improvement of two grades or more from the baseline, were achieved by 43.8% of patients receiving 200 mg abrocitinib, 29.6% of those receiving 100 mg, 8.9% of those receiving 30 mg, 10.9% of those receiving the 10 mg dose, and 5.8% of patients receiving placebo, respectively. Two treatment-related severe AEs occurred: one case of pneumoniae in the 200 mg abrocitinib group and one case of eczema herpeticum in the 100 mg abrocitinib group. Upper respiratory tract infection, headache, and nausea were the most frequent AEs. Decreased platelet counts were detected in the 200 mg and 100 mg abrocitinib groups but became normalized after 4 weeks of continuous treatment [22]. Maintenance of disease control during a 4-week drug-free follow-up period was assessed in a post hoc analysis [31]. Among the 200 mg abrocitinib responders, 77.4%, 42.3%, 21.1%, and 42.9% maintained their Eczema Area and Severity Index score (EASI)-50, EASI-75, Investigator’s Global Assessment (IGA), and pruritus Numerical Rating Score (pNRS) response at week 16, whereas for the 100 mg abrocitinib responders, the proportions were 51.9%, 35.0%, 33.3%, and 43.5%, respectively [31]. 

### 5.1. Abrocitinib vs. Placebo in Monotherapy: JADE MONO-1, MONO-2, and JADE REGIMEN

Phase 3 studies, JADE MONO-1 and JADE MONO-2, were two 12-week double-blind, placebo-controlled, parallel-group randomized clinical trials that assessed the efficacy and safety of abrocitinib monotherapy (100 or 200 mg dose) in patients aged 12 years or older [23,24]. Coprimary endpoints were met at 12 weeks, with a greater proportion of patients in the 100 and 200 mg abrocitinib groups achieving an improvement in the IGA response ≥2 grades and a 75% improvement in the EASI-75 vs. the placebo group. In the JADE MONO-1 trial, 23.7% and 39.7% of patients receiving 100 mg abrocitinib, 43.8% and 62.7% of participants receiving 200 mg abrocitinib, and 7.9% and 11.8% of subjects receiving placebo achieved an IGA response and an EASI-75 at week 12, respectively. Statistical significance in peak P-NRS (PP-NRS) response was noted as early as week 2 [23].

In the JADE MONO-2 trial, IGA and EASI-75 responses were superior for abrocitinib 100 mg and abrocitinib 200 mg vs. placebo (44 of 155 [28.4%] and 59 of 155 [38.1%] vs. 7 of 77 [9.1%]; *p* < 0.001) and (69 of 155 [44.5%] and 94 of 154 [61.0%] vs. 8 of 77 [10.4%]; *p* < 0.001), respectively. IGA and EASI-75 responses were observed since week two of treatment and were sustained until week 12. As for secondary endpoints, a greater estimated proportion of patients treated with abrocitinib achieved a PP-NRS response ≥4 (45.2% [95% CI, 37.1–53.3%] and 55.3% [95% CI, 47.2–63.5%] vs. 11.5% [95% CI, 4.1–19.0%]; *p* < 0.001) and greater proportions of patients in the active arms achieved at least 90% improvement in EASI score (EASI 90) responses (37 of 155 [23.9%] receiving abrocitinib 100 mg and 58 of 154 [37.7%] receiving abrocitinib 200 mg vs. 3 of 77 [3.9%]) receiving placebo). Interestingly, PP-NRS scores differed significantly between both doses of abrocitinib and placebo within 24 h of the first dose of treatment [24]. Furthermore, pooled data from previous trials (phase 2b, JADE MONO-1 and JADE MONO-2) showed that itch improvement was independent of itch severity, sex, race, body mass index, or IGA response [32].

Regarding AEs, there were no reports of malignancy, major cardiovascular AEs, or venous thromboembolisms in either of the studies. Serious AEs related to treatment included the development of inflammatory bowel disease in one patient in the 200 mg group, acute pancreatitis in one patient in the 100 mg group, herpangina in one patient in the 200 mg group, and pneumonia in the 100 mg group in JADE MONO-1 and 2 studies, respectively [24]. The most frequently reported AEs in JADE MONO-1 and MONO-2 were nausea in the 200 mg group (31% and 14.2%, respectively) and nasopharyngitis in the 100 mg group (15% and 12.7%). The incidences of herpes zoster and eczema herpeticum in both groups were low and comparable with those of the placebo group. There were no clinically significant changes in hemoglobin level, neutrophil, or lymphocyte counts. Increased lipids levels were observed, as well as dose-related decreases in median platelet counts that stabilized by week 12 without clinical sequelae [23,24]. 

In the phase 3 trial JADE REGIMEN, patients with moderate-to-severe AD responding to open-label abrocitinib 200 mg monotherapy for 12 weeks were randomly assigned to receive abrocitinib (200 or 100 mg) or a placebo n a 1:1:1 ratio for 40 weeks [27]. Those patients experiencing flares during treatment received additional rescue treatment with abrocitinib 200 mg plus topical therapy. After 12 weeks of open-label induction with 200 mg of abrocitinib in monotherapy, 798 patients (64.7%) achieved the IGA 0/1 and EASI-75 response defined by the protocol and were randomly assigned to the three above-mentioned arms into the maintenance period. After 40 weeks, the cumulative probabilities of experiencing a flare were 18.9% (95% CI, 14.2–24.9) in the abrocitinib 200 mg group, 42.6% (95% CI, 36.3–49.5) in patients receiving 100 mg of the active drug, and 80.9% in the placebo group. The median time to protocol-defined flare (according to the Kaplan-Meier estimator) was 28 days (95% CI, 28–29) in the placebo arm; it was not reached in either abrocitinib arm. After rescue treatment, 36.6%, 58.8%, and 81.6% of patients regained IGA 0/1 response, and 55.0%, 74.5%, and 91.8% regained EASI-75 response in the abrocitinib 200 mg, abrocitinib 100 mg, and placebo groups, respectively. Regarding safety, 6.0%, 1.9%, and 1.5% of patients discontinued treatment due to an AE in the abrocitinib 200 mg, 100 mg, and placebo maintenance groups, respectively. One patient in the 100 mg abrocitinib group experienced retinal vein thrombosis during the maintenance period. As in previous studies, increases in lipids and dose-related decreases in median platelet count were observed in abrocitinib-treated patients, with the nadir occurring at week 4 and posterior return to baseline values [27].

### 5.2. Abrocitinib vs. Dupilumab or Placebo with Topical Therapy: JADE COMPARE and JADE EXTEND

A phase 3 randomized, double-blind, placebo-controlled trial randomized 838 adult patients with moderate-to-severe AD to receive oral abrocitinib 200 or 100 mg once daily, dupilumab 300 mg subcutaneous injection every 2 weeks, or placebo (allocation ratios, 2:2:2:1), with topical background therapy (low- or medium-potency topical corticosteroids, topical calcineurin inhibitors, or topical phosphodiesterase-4 inhibitors) for 16 weeks [25]. By week 12, IGA and EASI-75 responses were observed in 48.4% and 70.3% of patients in the 200 mg abrocitinib group, 36.6% and 58.7% in the 100 mg abrocitinib group, 36.5% and 58.1% in the dupilumab group, and 14.0% and 27.1% in the placebo group, respectively (*p* < 0.001 for both abrocitinib doses vs. placebo). Regarding secondary endpoints, the 200 mg dose of abrocitinib was superior to dupilumab with respect to itch at week 2. No other significant differences were observed in key secondary end-point comparators between abrocitinib and dupilumab by week 16 [25]. Clinically meaningful improvements in selected patient-reported outcomes (PROs) showed statistically significant improvements at week 16 in both doses of abrocitinib vs. placebo [33]. Rapid and persistent improvement was observed with abrocitinib treatment: patients treated with abrocitinib 200 mg achieved EASI-75 response at a median of 29 days across body regions (including the head and neck region); in patients receiving abrocitinib 100 mg, EASI-75 response was achieved at a median of 30–32 days for trunk and lower limbs and at 56–57 days for the head and neck region and upper limbs; in the dupilumab group, this response was achieved at a median of 43 days for the trunk and 57 days for the other regions [34]. 

No deaths, major cardiovascular AEs, or thromboembolic events occurred during the trial [25]. The incidence of AEs was higher in the group receiving abrocitinib 200 mg than in the others. Nausea and acne were more frequent in the abrocitinib groups (11.1% and 6.6% for patients receiving the 200 mg dose, 4.2% and 2.9% for those receiving the 100 mg dose, 2.9% and 1.2% for patients treated with dupilumab and 1.5% and 0 for those receiving placebo, respectively). Conjunctivitis was reported more frequently in the dupilumab group (6.2% vs. 1.3% in the abrocitinib 200 mg group, 0.8% in the 100 mg group, and 2.3% in the placebo group). A total of 4 patients (1.8%) in the 200 mg abrocitinib group and 2 (0.8%) in the 100 mg group had herpes zoster infection, none of them involving dose changes nor withdrawal from the trial [25]. Dose-dependent decreases in median platelet counts, as well as increases in median creatine kinase level, mean total cholesterol level, and mean high and low-density lipoprotein cholesterol levels, were reported among the patients who received abrocitinib. Thrombocytopenia was reported in 2 patients (110,000 and 118,000 platelets/mm^3^) in the 200 mg abrocitinib group [25]. 

Results of the long-term extension study JADE EXTEND have been published [28]. In this randomized phase 3 study, patients from the JADE COMPARE trial who had received subcutaneous dupilumab until week 14 were given an oral placebo until week 20 and then entered in JADE EXTEND. Patients were randomly assigned to receive abrocitinib 200 mg or 100 mg once daily. The efficacy responses with abrocitinib at week 12 of JADE EXTEND were analyzed based on the prior dupilumab response status of patients at week 16 of JADE COMPARE. Among prior dupilumab IGA 0/1 and PP-NRS4 responders, 83.3% and 89.7% of abrocitinib 200 mg and 76.9% and 81.6% of abrocitinib 100 mg-treated patients achieved IGA 0/1 and PP-NRS4 responses at week 12 of the JADE EXTEND study. Among patients who achieved EASI-75 but not EASI-90 with dupilumab, 64.7% and 54.1% treated with abrocitinib 200 mg and 100 mg, respectively, achieved EASI-90 at week 12 following the switch. Regarding dupilumab non-responders, 47.2% and 77.3% of patients treated with abrocitinib 200 mg and 35.2%, 37.8% of those treated with abrocitinib 100 mg achieved IGA 0/1 and PPRNS responses, respectively. Moreover, among patients who did not achieve any of the efficacy responses (IGA 0/1, EASI-75, or PP-NRS4) with dupilumab in JADE COMPARE, 45.5% and 16.7% of patients treated in JADE EXTEND with abrocitinib 200 mg and 100 mg, respectively, gained the three responses by week 12 [28]. It is worth mentioning that in patients who switched from dupilumab to abrocitinib, after 6 weeks of placebo washout and thereafter, some residual effect of dupilumab might have contributed to the efficacy of abrocitinib in JADE EXTEND.

### 5.3. Abrocitinib with Topical Therapy in Teenagers: JADE TEEN

JADE TEEN was a phase 3 double-blind, placebo-controlled clinical trial that included 285 patients aged 12–17 years with moderate-to-severe AD, who were randomly assigned 1:1:1 to receive once-daily oral abrocitinib, 200 mg or 100 mg, or placebo for 12 weeks in combination with topical therapy [26]. Coprimary endpoints were IGA (0/1) response and EASI-75 response at week 12. IGA (0/1) response was achieved by 43 (46.2%), 37 (41.6%), and 23 (24.5%) patients in the abrocitinib 200 mg, abrocitinib 100 mg, and placebo groups, respectively (*p* < 0.05 for both abrocitinib doses); the corresponding EASI-75 response figures were 67 (72.0%), 61 (68.5%), and 39 (41.5%) patients (*p* < 0.05 for both). Differences in PP-NRS scores between the abrocitinib groups vs. placebo were observed within 2 days of dose 1. Improvements in quality of life were assessed with PROs, with greater improvements for the abrocitinib groups in both patients and caregivers [26,35].

AEs were reported in 62.8%, 56.8%, and 52.1% of patients in the 200 mg, 100 mg, and placebo groups, respectively. They led to discontinuation in two (2.1%), one (1.1%), and two (2.1%) patients, respectively. Serious AEs were reported in two (2.1%), 0, and two (2.1%) patients in the abrocitinib 200 mg, 100 mg, and placebo groups. Nausea was more common with abrocitinib 200 mg; the median times to the onset of nausea were 2.0, 4.0, and 1.0 days in patients treated with abrocitinib 200 mg, abrocitinib 100 mg, and placebo, respectively; the median times to resolution were 13.0, 23.0, and 1.0 days, respectively. Cases of serious infection, venous thromboembolism, cancers, major cardiovascular AEs, or deaths were not reported [26]. Modest increases in serum levels of total cholesterol and high- and low-density lipoprotein were detected for both doses of abrocitinib vs. placebo; they reached a plateau at week 4. As in previous studies, abrocitinib-treated patients experienced dose-related decreases in median platelet cell counts, with a nadir at week four and a return toward baseline values thereafter [26]. 

### 5.4. Abrocitinib vs. Dupilumab with Topical Treatment: JADE DARE

JADE DARE was a phase 3 randomized, double-blind, active-controlled, parallel-treatment clinical trial in which adults with moderate-to-severe atopic dermatitis were randomized to receive abrocitinib 200 mg or dupilumab 300 mg (every 2 weeks) for 26 weeks [29]. Coprimary endpoints (reaching PP-NRS4 by week two and EASI-90 by week four) were met by a higher proportion of abrocitinib-treated patients as compared to dupilumab-treated ones (48% vs. 26% and 29% vs. 15%, respectively). One of the key secondary endpoints, EASI-90 response at 16 weeks, was met by 54.3% in the abrocitinib group vs. 41.9% in the dupilumab group [29]. 

Regarding safety, AEs were more frequent in the abrocitinib (74%) than in the dupilumab group (65%). Nausea, headache, acne and folliculitis, and conjunctivitis were the most frequent AEs, occurring in more than 5% of patients for both treatment groups. All of them were more frequent in abrocitinib except conjunctivitis (19% vs. 2%, 13% vs. 7%, 13% vs. 11%, and 3% vs. 11%, respectively). Nausea led to discontinuation in two patients and headache in one patient, all in the abrocitinib group. Herpes simplex infections occurred in 6% of the abrocitinib-treated patients and 5% of dupilumab-treated ones. Herpes zoster was reported in 2% of abrocitinib patients and <1% of dupilumab. No malignancy or venous thromboembolic events were observed in either group [29].

### 5.5. Other Studies

Results from an integrated safety analysis based on long-term follow-up of patients from the phase 2 and phase 3 clinical trials, including 2856 patients and corresponding to a total of 1614 patient-years (PY), have been published [36]. Total exposure in the pooled abrocitinib cohort was ≥24 weeks in 1248 patients and ≥48 weeks in 606; the maximum duration of follow-up was 108 weeks. The most commonly reported AEs for abrocitinib 100 mg, abrocitinib 200 mg, and placebo were nausea (6.1%, 14.6%, and 2.0% of patients, respectively), headache (5.9%, 7.8%, and 3.5%, respectively), and acne (1.6%, 4.7%, and 0%, respectively) [21]. In patients treated with abrocitinib 100 mg and 200 mg, the incidence rates (IR) of herpes simplex were 8.73 per 100 PY and 11.83 per 100PY, respectively, and those of herpes zoster 2.04 per 100 PY and 4.34 per 100 PY, whereas the IR of serious infection was 2.65 per 100 PY and 2.33 per 100 PY, respectively [21]. There were five events of venous thromboembolism (VTE) (IR 0.30/100 PY), all in the 200 mg group. Three serious AEs were adjudicated as pulmonary embolism (PE): in a 55-year-old white male with asthma, a 68-year-old white female receiving estrogen treatment, with a history of uterine prolapse, menopause, hypertension, hypercholesterolemia, and first-degree atrioventricular block, and a 16-year-old black male with morbid obesity and an extensive family history of PE. The IR for PE was 0.18/100 PY (95% CI 0.04–0.52). Two AEs were adjudicated as deep venous thrombosis (DVT): a 44-year-old white female who experienced calf thrombosis after arthroscopic surgery and a 50-year-old Asian female with obesity and hypertension who experienced superficial thrombophlebitis in the left great saphenous vein and left superficial femoral vein. The IR for DVT was 0.12/100 PY (95% CI 0.01–0.43) [21]. Exclusion criteria for patients with a high risk of VTE, such as previous PE or recent hospitalization, were eventually added to the abrocitinib development program as the class risk of JAK inhibitors for VTE became clearer. The incidence rates of nonmelanoma skin cancer, other malignancies, or cardiovascular events were very low (less than 0.5 cases per 100 PY) [21].

A post hoc preliminary economic analysis of abrocitinib treatment for moderate-to-severe AD used data from JADE COMPARE and JADE MONO-2. Preliminary results suggest that treatment with abrocitinib decreases physician visits, work impairment, and costs associated with management compared to placebo [37].

Two ongoing clinical trials are further investigating abrocitinib in AD: NCT03915496 (phase 2a) evaluates the mechanism of action of the drug in AD patients [38], and NCT04564755 (phase 3) is an expanded access protocol for treatment in adolescents and adults [39]. Furthermore, two post-marketing surveillance trials to evaluate real-world data are currently ongoing (NCT05391061, NCT05250115) [40,41].

### 5.6. Comparison with Other AD Therapies 

A recent network meta-analysis (NMA) including 11 clinical trials with 6254 patients has compared the efficacy of several approved treatments for AD: upadacitinib 30 mg, upadacitinib 15 mg, abrocitinib 200 mg, abrocitinib 100 mg, baricitinib 4 mg, baricitinib 2 mg, dupilumab 300 mg, and tralokinumab 300 mg [42]. Monotherapy with abrocitinib 200 mg daily obtained the second highest efficacy at the primary endpoint evaluation (EASI-75, EASI-90, NRS ≥ 4), being first upadacitinib 30 mg [42]. The EASI-75 response rate estimates at the primary endpoint timepoints of the clinical trials were 70.8% (95% Credible Interval [CrI], 54.7–83.0%) for upadacitinib 30 mg, 62.9% (95% CrI 42.5–79.9%) for abrocitinib 200 mg, 58.1% (95% CrI 40.9–73.5%) for upadacitinib 15 mg, 43.5% (95% CrI 27.4–61.0%) for dupilumab 300 mg, 43.0% (95% CrI 24.8–64.0%) for abrocitinib 100 mg, 34.1% (95% CrI 19.4–52.6%) for baricitinib 4 mg, 29.6% (95% CrI 16.8–46.8%) for baricitinib 2 mg, and 27.8% (95% CrI 15.9–43.9%) for tralokinumab 300 mg, with 11.3% (95% CrI 6.3–19.2%) for placebo. Of note, most of the trials had up to 14-week follow-up periods, lacking long-term efficacy studies much needed in chronic conditions. 

Another NMA has been conducted on the safety of JAK inhibitors in AD, including 18 randomized clinical trials [43]. Results showed that JAK inhibitors present comparable safety profiles; statistically significant differences were observed only when compared with placebo or dupilumab [43]. Baricitinib, abrocitinib, and upadacitinib increased the risk of any AE compared with placebo; the corresponding odds ratios (OR) were 1.25 (95% CrI 1.03–1.55), 1.54 (95% CrI 1.25–1.90), and 1.46 (95% CrI 1.19–1.81), respectively [43]. Abrocitinib, upadacitinib, and dupilumab increased the risk of infections compared to placebo: the corresponding OR were 1.62 (95% CrI 1.7–2.72), 1.67 (95% CrI 1.19–2.43), and 1.69 (95% CrI 1.02–2.79), respectively [43]. No statistically significant risk differences were identified between treatments regarding serious infections, but dupilumab was found to be numerically safest, followed by baricitinib and placebo [43]. In addition, conjunctivitis, head and neck erythema, and common AEs with dupilumab treatment have not been associated with JAK inhibitors [5].

## 6. Discussion

The development of targeted therapies in AD has addressed the pathogenic routes involved in this complex disease with enhancements in efficacy and safety versus classic systemic agents such as cyclosporin [44]. Approval of the IL-4 receptor inhibitor dupilumab revolutionized disease management, but there are some unmet needs. Dupilumab-associated conjunctivitis can usually be managed with ophthalmologic treatment, but some patients must interrupt dupilumab due to the development of AEs (mainly conjunctivitis and head and neck erythema). Primary non-responders to dupilumab, as well as those patients who cease to respond over time, need alternative therapies. This is specifically relevant in Asian populations where polarization towards the Th17 pathway has been demonstrated [45,46]. Furthermore, a specific subset of patients might require a more flexible dosing regimen according to individual signs and symptoms. Finally, there are selected patients who will not accept or tolerate the subcutaneous injection route. Therefore, new treatments are needed. The recent approvals of the IL-13 receptor inhibitors tralokinumab and lebrikizumab, the oral JAK inhibitors upadacitinib, abrocitinib, and baricitinib, and the ongoing development of anti-IL31 nemolizumab, may fulfill some of these unmet needs [47]. 

Abrocitinib decreases pruritus very rapidly and effectively, and itch relief was considered the most important factor in determining treatment response by AD patients [48]. Moreover, improvements in pruritus contribute to better sleep and further increase the quality of life in these patients [49]. JAK1 inhibition represents a good therapeutic strategy for patients suffering from significant pruritus and patients with an intense itch that does not respond to IL-4 receptor inhibitors, IL-13 inhibitors, and IL-31 receptor inhibitors [50]. 

Flexible dosing might be a valid option with abrocitinib and was assessed in the JADE REGIMEN clinical trial [27]. According to the results of this trial, abrocitinib 200 mg monotherapy was the most effective option for maintaining disease control, but the induction-maintenance approach with abrocitinib 200 mg followed by 100 mg might be adequate since most patients did not flare for at least 40 weeks [27]. In the trial, intermittent therapy with treatment discontinuation after obtaining a response gave a high relapse rate, so it is not recommended. Identification of predictors for maintaining clinical response is needed in order to establish an intermittent approach [27].

Currently, the approved indication of abrocitinib is the treatment of moderate-to-severe AD in adults who are candidates for systemic therapy. Cibinqo^®^ is marketed as 50 mg, 100 mg, and 200 mg film-coated tablets. The recommended starting dose is 200 mg once daily, but 100 mg is recommended for patients ≥ 65 years of age. Discontinuation of treatment should be considered in patients with no evidence of therapeutic benefit after 24 weeks.

Abrocitinib is subject to drug interactions with inhibitors or inducers of CYP2C19 or CYP2C9; dose adjustment is not required in patients with mild renal impairment (estimated glomerular filtration rate of 60 to <90 mL/min) or mild or moderate hepatic impairment. The potential for drug interactions and the need for dose adjustment in patients with significant renal or hepatic impairment are shared to some extent by all JAK inhibitors and may determine the choice of monoclonal antibodies for the treatment of AD in some patients.

Abrocitinib is contraindicated in patients with hypersensitivity to the active substance or any of the excipients, those with active serious systemic infections, including tuberculosis, and patients with severe hepatic impairment, as well as pregnant and breast-feeding women since studies in animals have shown reproductive toxicity.

Patients must be screened for latent tuberculosis (if positive, preventive therapy should be started prior to initiation of treatment) and viral hepatitis. Use of live, attenuated vaccines should be avoided during or immediately prior to treatment. Before starting treatment with abrocitinib (and other JAK inhibitors), it is recommended to update the immunization status of all patients, including prophylactic herpes zoster vaccinations (with recombinant Shingrix^®^) in adults 50 years or older, in accordance with official recommendations.

Common AEs of abrocitinib include self-resolving nausea, acne, nasopharyngitis, headache, upper respiratory infection, and treatment-emergent herpes viral infections; in most cases, they do not require permanent interruption of treatment and may be prevented or attenuated using lower doses without detriment to the efficacy of treatment.

Safety has become an issue with JAK inhibitors after both the FDA and EMA raised concerns for increased risk of major adverse cardiovascular events (MACE), malignancy, DVT/PE, and mortality. This is due to the detection of increased rates of DVT/PE and MACE in patients treated with tofacitinib and baricitinib for rheumatoid arthritis (RA) [51]. Of note, the risk of cardiovascular disease is increased among patients with RA owing to systemic inflammation [52]. Post hoc analyses of the open-label safety trial of tofacitinib in RA reveal that a history of DVT/PE, smoking, age >65 years, and hormone replacement therapy or oral contraceptive use significantly increase the risk of DVT/PE or MACE [53,54]. The risk associated with relatively more selective JAK inhibitors, such as upadacitinib and abrocitinib, in younger and healthier populations of AD patients, is expectedly lower. Anyhow, the EMA Pharmacovigilance Risk Assessment Committee (PRAC) has recommended that JAK inhibitors should be used only if no suitable treatment alternatives are available in patients aged 65 years or older, those at increased risk of major cardiovascular problems such as heart attack or stroke, patients who smoke or have been smokers for a long time, and those at increased risk of cancer. These recommendations apply to all approved uses of JAK inhibitors in chronic inflammatory disorders (RA, psoriatic arthritis, juvenile idiopathic arthritis, axial spondylarthritis, ulcerative colitis, AD, and alopecia areata) [55]. 

A recent metanalysis has assessed the association of incident DVT/PE with AD and treatment with JAK inhibitors (abrocitinib, baricitinib, upadacitinib, and ivarmacitinib), dupilumab, or placebo in RCTs [36]. With a total of 466,993 patients included in two cohort studies and 15 RCTs, no significant association of AD with incident DVT/PEV has been found (HR, 0.95; 95% CI 0.62–1.45; the incidence rate of venous thromboembolism was 0.23 events/100 PY). The meta-analysis found no significant difference in the risk of VTE between participants in RCTs receiving JAK inhibitors and controls receiving placebo or dupilumab: overall, 3 of 5722 (0.05%) patients with AD who were being treated with JAK inhibitors experienced DVT/PEV compared with 1 of 3065 (0.03%) patients with AD receiving placebo or dupilumab (Mantel-Haenszel risk difference, 0; 95% 95%CI, 0–0) [36]. Three DVT/PEV events occurred in participants who were receiving treatment with JAK inhibitors during the randomized placebo-controlled periods: a patient receiving abrocitinib 100 mg developed a retinal vein thrombosis [27], another patient with abrocitinib 200 mg developed a PE (but it was considered as not related to treatment) [22] and a patient receiving baricitinib 4 mg developed another PE [56]. Of note, patients with known coagulopathy or platelet dysfunction were excluded from phase 3 trials of abrocitinib (MONO-1 and MONO-2, COMPARE) [23,25].

Abrocitinib should be used with caution in patients at high risk for DVT/PE. Risk factors that should be considered in determining this risk include older age, obesity, a medical history of DVT/PE, prothrombotic disorder, combined hormonal contraceptives or hormone replacement therapy, and major surgery or prolonged immobilization. 

Laboratory abnormalities in AD patients under treatment with abrocitinib are very rare but have been reported in detail [21]. There was a dose-dependent decrease in platelets in the placebo-controlled cohort, with median values reaching a nadir at week four. Most patients (>95%) maintained platelet value > 100 × 10^3^/mm^3^, but nine patients in the 200 mg group had confirmed platelet values < 75 × 10^3^/mm^3^ between week two and month one of exposure; all patients but one were ≥65 years of age. In the all-abrocitinib cohort, there were no changes over time in median absolute lymphocyte count, absolute neutrophil count, or hemoglobin values, but 4 of 2832 (0.1%) patients met discontinuation criteria for lymphocytes (absolute lymphocyte count < 0.5 × 10^3^/mm^3^), all in the 200 mg group. These four patients were ≥65 years of age, and events in three patients occurred in the first four treatment weeks. Thus, laboratory screening is required before starting treatment with abrocitinib: it should not be initiated in patients with a platelet count < 150 × 10^3^/mm^3^, an absolute lymphocyte count < 0.5 × 10^3^/mm^3^, an absolute neutrophil count < 1.2 × 10^3^/mm^3^ or who have a hemoglobin value < 10 g/dL. Complete blood counts should be monitored 4 weeks after initiation of therapy and thereafter according to routine patient management. Dose-dependent increases in blood lipid parameters, specifically LDL cholesterol, have been reported in AD patients treated with abrocitinib, but there was no notable change in LDL cholesterol/HDL cholesterol ratio over 16 weeks; thus, lipid parameters should also be assessed approximately 4 weeks after initiation of therapy and thereafter according to the cardiovascular disease risk of each patient. A dose-related increase in blood creatine phosphokinase was observed with abrocitinib, but no cases of rhabdomyolysis were reported, and monitoring of creatine phosphokinase levels is not required.

In summary, JAK inhibitors-associated AEs seem to be dose-dependent. Specifically, for abrocitinib, fewer AEs were reported in patients receiving abrocitinib 100 mg versus those receiving abrocitinib 200 mg [23,25,27].

## 7. Conclusions

Treatment with abrocitinib has been demonstrated to be efficacious and safe in clinical trials with selected AD patients. Improvement in itch was noticeable within 24 h after administration, and efficacy was comparable to dupilumab. Being a small molecule that can be administered orally, abrocitinib is more suitable for patients willing to avoid injections. Adequate monitoring and consideration of use in patients with cardiovascular disease, a history of thrombotic events, and a history of herpes infection will be necessary to minimize safety concerns. Long-term follow-up studies will better define the safety profile of the JAK1 inhibitor abrocitinib in AD and contribute to the best exploitation of this significant therapeutic advance.

## Figures and Tables

**Table 1:** Summary of published data from abrocitinib clinical trials. IGA: Investigator Global Assessment. EASI: Eczema Area and Severity Index; pNRS (pruritus Numerical Rating Score); AEs: Adverse Events.

Trial	Design	Dose Regimen	Patients	Study Endpoints	Efficacy	Adverse Effects
NCT02780167	Randomized, double-blind, placebo-controlled, parallel-group phase 2b trial	Abrocitinib 200 mg Abrocitinib 100 mg Placebo	N = 267	Efficacy (IGA, EASI) Safety and tolerability	IGA response in 43.8% abrocitinib 200 mg and 29.6% abrocitinib 100 mg (*p* < 0.001) EASI response in 82.6% abrocitinib 200 mg and 59.0% abrocitinib 100 mg (*p* < 0.001)	AEs in 184 patients (68.9%). Most frequent were upper respiratory infections, headache, nausea, and diarrhea
NCT03349060 (JADE MONO 1)	Multicenter, double-blind, randomized phase 3 trial 12 weeks	Abrocitinib 200 mg Abrocitinib 100 mg Placebo	N = 387	Efficacy (IGA, EASI) Safety and tolerability	IGA response in 63% (*p* = 0.0037) abrocitinib 200 mg and 44% (*p* < 0.0001) in abrocitinib 100 mg EASI response in 24% and 40% of abrocitinib 200 and 100 mg (*p* < 0.0001), respectively	Most frequent AEs included nausea (9% and 20%), nasopharyngitis, headache, upper respiratory infection Serious AE occurred in 4% of patients
NCT03422822 (JADE EXTEND)	Long-term extension, randomized, phase 3 trial 12 weeks	Abrocitinib 200 mg Abrocitinib 100 mg	N = 223	Efficacy of abrocitinib following dupilumab Safety and tolerability	IGA 0/1, EASI-75, and PPRNS responses were obtained in 45.5% and 16.7% of the 200 mg and 100 mg abrocitinib groups (dupilumab non-responders)	The most frequent AEs were nasopharyngitis, nausea, acne, and headache.
NCT03575871 (JADE MONO 2)	Double-blind placebo-controlled, parallel, parallel-group, randomized phase 3 trial 12 weeks	Abrocitinib 200 mg Abrocitinib 100 mg Placebo	N = 391	Efficacy (IGA, EASI, ppNRS) Safety and tolerability	IGA response in 48.4% and 36.6% in abrocitinib 200 mg and 100 mg, respectively. EASI-75 response in 61% and 44.5% of abrocitinib 200 mg and 100 mg, respectively.	AEs occurred in 62.7%, 65.8%, and 53.8% of patients with abrocitinib 200 mg, 100 mg, and placebo, respectively
NCT03627767 (JADE REGIMEN)	Multicenter, responder-enriched, double-blinded, placebo-controlled, phase 3 randomized withdrawal trial with rescue treatment 52 weeks	Abrocitinib 200 mg Abrocitinib 100 mg Placebo	N = 1233	Efficacy of rescue therapy following dose reduction of withdrawal of abrocitinib Safety and tolerability	After 40 weeks of the maintenance period, the probability of experiencing a flare was 18.9%, 42.6%, and 80.9% in the abrocitinib 200 mg, 100 mg, and placebo groups, respectively. After rescue treatment, 36.6%, 58.8%, and 81.6% regained IGA 0/1 response, and 55.0%, 74.5%, and 91.8% regained EASI-75 response in abrocitinib 200 mg, 100 mg, and placebo groups, respectively.	AEs were experienced in 63.2% and 54% of patients in the 200 mg and 100 mg abrocitinib group during the second phase of the study.
NCT03720470 (JADE COMPARE)	Multi-center, randomized, double-blind, double-dummy, placebo-controlled phase 3 trial 16 weeks	Abrocitinib 200 mg Abrocitinib 100 mg Dupilumab Placebo	N = 838	Efficacy (IGA, EASI, NRS) Safety and tolerability	IGA response in 48.4% of abrocitinib 200 mg, 36.6% of abrocitinib 100 mg, 36.5% of dupilumab, and 14% of placebo. EASI-75 response was achieved for 70.3%, 58.7%, 58.1%, and 27.1% for abrocitinib 200 mg, 100 mg, dupilumab, and placebo, respectively.	AEs were experienced in 61.9%, 50.8%, 50.0%, and 53.4% of abrocitinib 200 mg, 100 mg, dupilumab, and placebo groups, respectively. Main AEs were nausea, acne, nasopharyngitis, headache
NCT03796676 (JADE TEEN)	Randomized, double-blind, placebo-controlled phase 3 trial 12 weeks	Abrocitinib 200 mg Abrocitinib 100 mg Placebo	N = 273	Efficacy (IGA, EASI, pNRS) Safety and tolerability	IGA and EASI-75 responses were achieved in 46.2%, 41.6%, and 24.5% and 72.0%, 68.5%, and 41.5% of abrocitinib 200 mg, 100 mg, and placebo, respectively.	AEs were experienced in 62.8%, 56.8%, and 52.1% of patients with abrocitinib 200 mg, 100 mg, and placebo, respectively. Nausea was the most common AE.
NCT04345367 (JADE DARE)	Randomized, double-blind, placebo-controlled phase 3 trial 26 weeks	Abrocitinib 200 mg Dupilumab 300 mg	N = 728	Efficacy (pNRS, EASI-90) Safety and tolerability	PP-NRS response was achieved at week 2 by 48.2% and 25.5% in abrocitinib and dupilumab groups, respectively EASI-90 at weeks 4 and 16 was achieved by 54.3% vs. 41.9% in the abrocitinib and dupilumab groups, respectively.	AEs were experienced by 74% and 65% of patients with abrocitinib and dupilumab, respectively. Nausea, headache, acne and folliculitis, and conjunctivitis were the most common AEs in both groups.

## Data Availability

Not applicable.
